# A retrospective stenting study on superior vena cava syndrome caused by lung cancer

**DOI:** 10.1111/1759-7714.13461

**Published:** 2020-05-21

**Authors:** Sen Wei, Jinghao Liu, Xin Li, Zuoqing Song, Ming Dong, Honglin Zhao, Qingchun Zhao, Gang Chen, Jun Chen

**Affiliations:** ^1^ Department of Lung Cancer Surgery, Tianjin Key Laboratory of Lung Cancer Metastasis and Tumor Microenvironment, Tianjin Lung Cancer Institute Tianjin Medical University General Hospital Tianjin China

**Keywords:** Endovascular stenting, lung cancer, prognosis, superior vena cava syndrome

## Abstract

**Background:**

Superior vena cava syndrome (SVCS) is a common condition predominantly caused by lung cancer. The presence of symptoms of SVCS, such as elevated intracranial pressure and laryngeal edema, indicates an unfavorable prognosis for lung cancer patients. Superior vena cava (SVC) stenting is the first‐line treatment for SVCS. In this study, we retrospectively analyzed SVCS cases treated with stenting in our center to explore the safety and effectiveness of stenting in the treatment of SVCS.

**Methods:**

We reviewed 16 patients with SVCS caused by lung cancer who were treated at our center with endovascular stenting between 2016 and 2018. Patient information such as age, sex, type of lung cancer, obstruction condition, complications, survival time, and postoperative treatments are summarized.

**Results:**

There were no treatment‐related complications in the perioperative period in any of the patients. Examination at postoperative day 2 indicated that the accompanying SVCS symptoms had improved in all patients. The median survival of patients treated along with combined postoperative chemotherapy and antivascular targeted therapy reached seven months (1–18 months).

**Conclusions:**

SVC stenting is effective as a first‐line treatment modality for patients with SVCS caused by lung cancer. In combination with other treatment modalities, it can significantly alleviate symptoms and reduce complications, and thus it plays an important role in the treatment of patients with SVCS caused by lung cancer.

## Introduction

The superior vena cava (SVC), which is formed by the left and right brachiocephalic veins, enters the right atrium along the left side of the trachea and aorta. The SVC has an average length of 1.4 ± 7.1 cm and an average diameter of 0.7 ± 2.1 cm in adults.^1^ Computed tomography (CT) scan examinations indicate that the diameter of the SVC major axis normally ranges from 1.5 cm to 2.8 cm and for the minor axis is 1.0 to 2.4 cm, and that an SVC area less than 1.07 cm^2^ is regarded as obstructed.^2^

Superior vena cava syndrome (SVCS) may have one or more possible etiologies, including compromised vessel anatomy, compromised vessel wall integrity, and compromised venous flow,^3^ all of which may be congenital, but are more likely to be acquired.^4^ The first documented SVCS case was reported by William Hunter in 1757.^5^ The etiology of SVCS has changed over the past few centuries; untreated or poorly treated infectious diseases such as tuberculosis and syphilis were formerly the major causes, but these days most cases are caused by malignant tumors.^6^, ^7^ Indeed, up to 70%–90% of SVCS cases are now caused by malignant tumors through compression of the SVC.^8^, ^9^ However, any disease that may cause right upper mediastinal and/or right paratracheal lymphadeopathy can lead to SVCS.^10^ The most common type of cancer associated with SVCS is lung cancer,^11^, ^12^ but others include bronchial malignancies, lymphomas, metastatic malignancies, thymoma, and mesothelioma.^11^ Some researchers believe that among bronchial lung cancers, non‐small cell lung cancer (NSCLC) is most likely to cause SVCS.^11^ However, Rowel *et al*. analyzed a series of cases and concluded that small cell lung cancer (SCLC) has a higher probability of causing SVCS than does NSCLC, with only 1.7% of NSCLC patients developing SVCS while 10.0% of SCLC patients developed SVCS.^13^ This is because in SCLC, not only the tumors tend to form masses and progress rapidly, but also the paratracheal lymph nodes like to fuse, and together they act to compress the SVC. However, because SCLC only accounts for 15% of the total incidence of lung cancer, SVCS caused by SCLC only accounts for 22% of the overall SVCS cases caused by malignant tumors.^14^ It is noteworthy that the implantation of intravascular devices has also become a cause of SVCS, accounting for over 40% of SVCS cases not caused by neoplastic diseases.^15^, ^16^, ^17^ Endothelial injury or stimulation of the SVC endothelium by the tip of the catheter in these cases cause inflammation and fibrosis, which will lead to VC stenosis.^18^, ^19^, ^20^ Rossi *et al*. found that 25% of patients with pacemakers had central venous obstruction or stenosis.^21^ However, only 1%–3% were symptomatic, presumably due to the slow progression of the obstruction and the concurrent formation of collateral circulation pathways.

### Patients treated in our center

Using LinkDoc data analysis, we summarized the treatment and prognosis of a total of 16 patients with SVCS caused by lung cancer who underwent stenting in our center between 2016 and 2018. Of these, 14 were men and two were women. Specific clinical data are summarized in Table 1. This study was approved by the Ethical Review Committee of Tianjin Medical University General Hospital. All biological samples and images were obtained with patients' written informed consent.

**Table 1:** The clinical characteristics of lung cancer patients with superior vena cava syndrome (SVCS)

Characteristics	Number
Age (years)	
<60	3
≥60	13
Gender	
Male	14
Female	2
Cause of SVCS	
Adenocarcinoma	10
Squamous cell carcinoma	2
Adenocarcinoma with neuroendocrine carcinoma	1
Small cell lung cancer	3
Treatments after stenting	
Chemotherapy	12
Chemotherapy with target therapy	4
Overall survival since SVCS diagnosis (months)	7 (1–18)

Symptomatic SVCS patients were at high risk of undergoing tracheoscopy because of severe coughing that would consequently elevate intracranial pressure. Therefore, CT‐guided percutaneous lung biopsy or superficial lymph node biopsy was performed instead to determine the pathology. Pathologic classifications of the patients revealed 11 cases of lung adenocarcinoma, three cases of small cell lung cancer, and two cases of lung squamous cell carcinoma. Furthermore, 12 cases with simple SVC construction and four cases with SVC combined right brachiocephalic vein obstructions were diagnosed by preoperative enhanced CT. Because all 16 patients exhibited certain clinical manifestations of SVCS, stenting was adopted as the first‐line treatment to alleviate symptoms. Stent implantation has gradually become the first‐line treatment for patients with SVCS,^16^, ^22^ and in order to ensure the effective treatment of patients, we did not use traditional radiotherapy and chemotherapy as the first‐line treatment.

**Figure 1:**
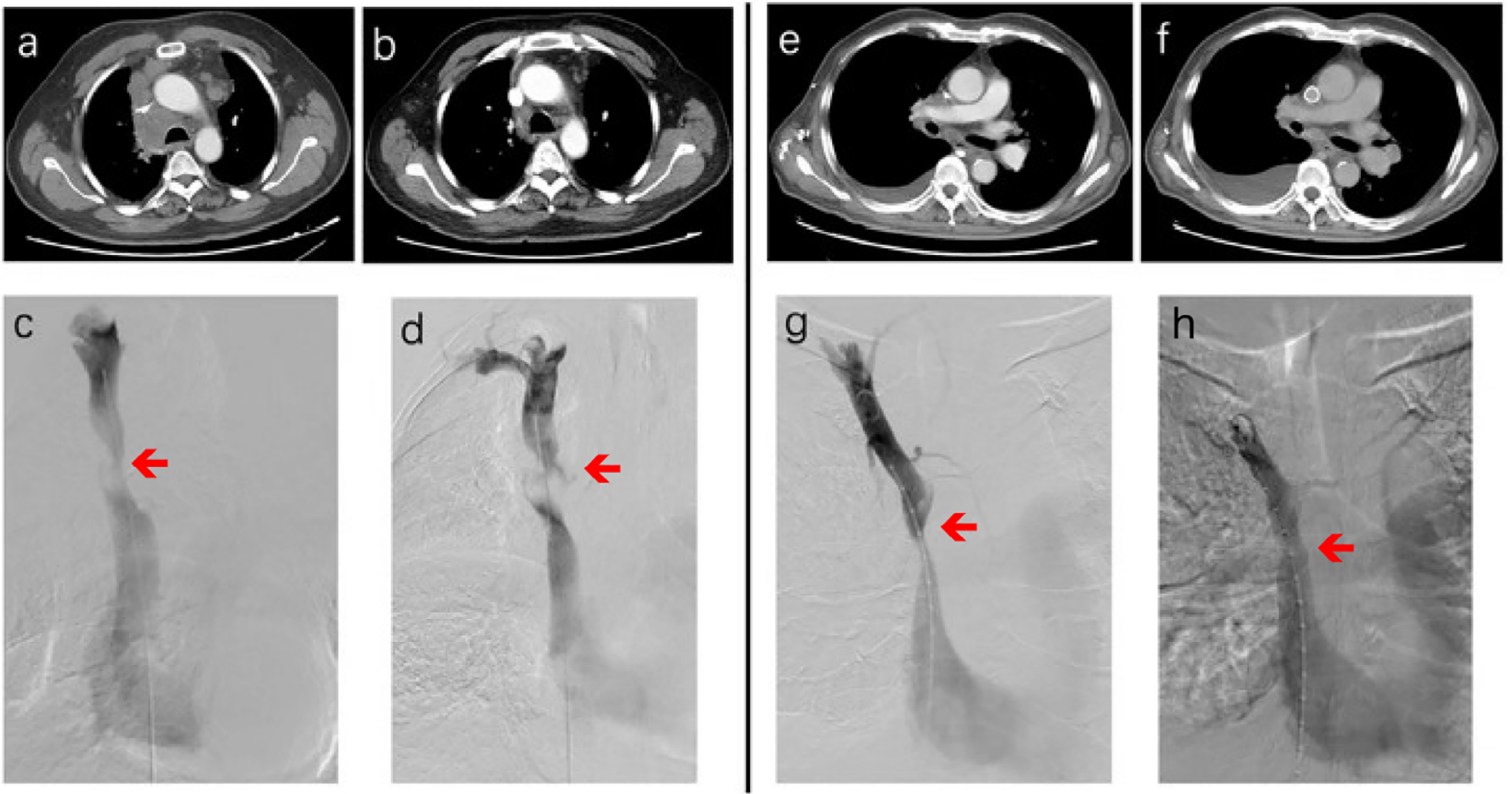
The pre‐ and postoperative images of the two patients with superior vena cava syndrome (SVCS). (**a**–**d**) Patient 1 and (**e**–**f**) patient 2. (**a** and **e**) Preoperative computed tomography (CT) scan images of both patients; and (**b** and **f**) postoperative CT scan images of both patients. (**c** and **g**) Preoperative angiography of both cases; and (**g** and **h**) postoperative angiography of both cases.

**Figure 2:**
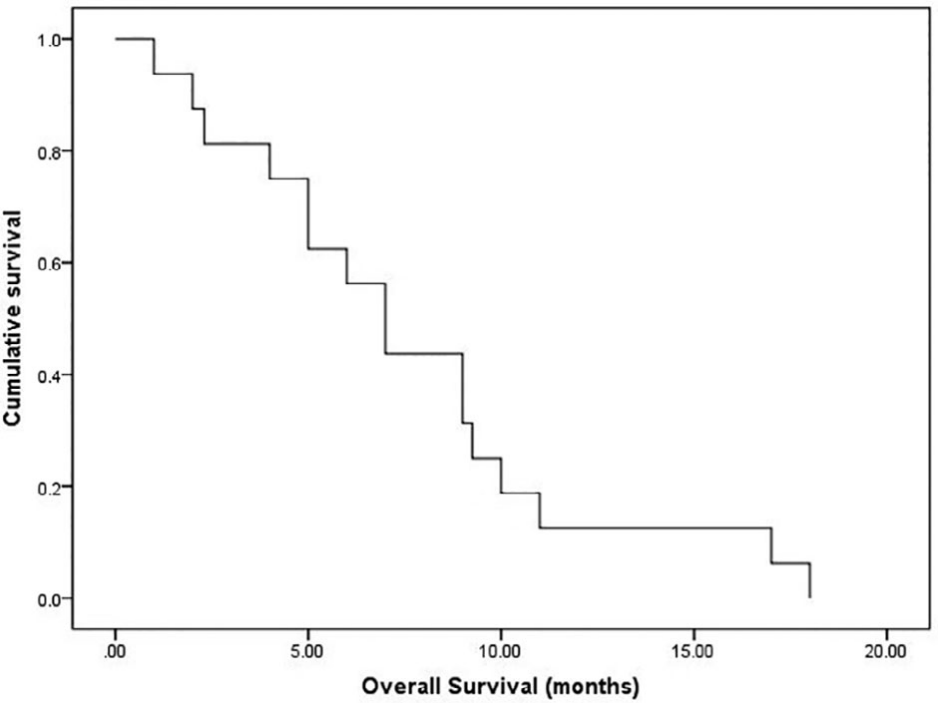
The overall survival for all patients. The median survival of all patients was seven months (1–18 months).

**Figure 3:**
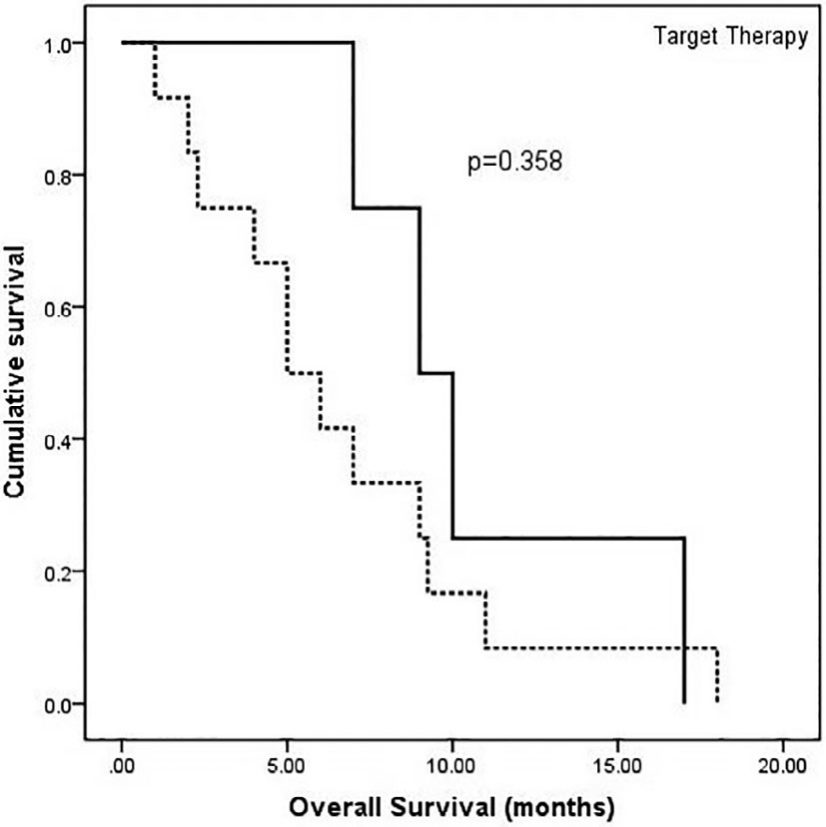
Survival curves of two groups based on treatment of patients with or without antivascular targeted drugs. There was a benefit tendency for the group receiving targeted drugs after chemotherapy, but the overall survival between these two groups did not have a significant difference (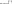) No, (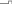) Yes.

The stent was implanted following routine protocol as follows. After skin preparation and draping, a catheter was introduced into the right femoral vein. Angiography was performed when the catheter arrived at the distal end of the SVC. Once the location of the stenosis was identified, the super‐lubricant super‐hard guidewire‐WALLSTENT was placed in situ. To ensure that the strength of the stent is supported, the hard part of the guidewire should travel across the stenosis, and therefore the guidewire catheter was placed approximately 10 cm beyond the stenosis. No obvious complications were observed during surgery. Notwithstanding the current discussions on postoperative anticoagulant therapy,^23^ all patients who underwent stent placement were administered long‐term anticoagulant agents to ensure the patency of the stents after operation. In addition, all patients received platinum‐based two‐drug chemotherapy after stenting, and in the case of the four patients who completed the planned chemotherapy regimen, antivascular targeted agent (anlotinib, a new small molecule multitarget tyrosine kinase inhibitor, which can effectively inhibit VEGFR, PDGFR, FGFR, c‐Kit and other kinases, with antitumor angiogenesis and antitumor growth) was also subsequently given. All patients experienced alleviation of their symptoms within two days of the operation. The images of the two cases who underwent stenting are compared in Fig 1. After stenting, the patients received the opportunity of further treatments with good effects. Due to the large time span, we were unable to collect detailed data on reblockage of the stent. The overall survival flow for these 16 patients is shown in Fig 2, and the median survival of all patients was seven months (1–18 months). In addition, we also analyzed the overall survival in these two groups of patients based on whether the targeted drug was used or not. As shown in Fig 3, the survival curves between these two groups with or without anlotinib were separated, but there was not a significant difference in overall survival. There may only have been a benefit tendency for the group with targeted drugs after chemotherapy.

## Discussion

When reviewing our data, we noted that men accounted for the majority of the patients with SVCS caused by lung cancer (89%). This result was similar to the reports of Kuo *et al*.^24^ and Gwon *et al*.^25^ in which the proportion of male patients was higher than that of females at 100% and 89%, respectively. However, whether this observation comes from data bias due to the small sample size or from the nature of the disease requires further verification. In addition, because the cases at our center were relatively simple, and no patients with SVC obstruction who were complicated with left and right brachiocephalic vein obstruction were treated, the procedure was uneventful and none of the common complications occurred, such as intraoperative bleeding and short‐term stent occlusion or misplacement.^26^

Our study observed a median survival of seven months for all patients, which was consistent with the six‐month survival of similar patients reported in previous studies.^4^, ^27^ However, compared to patients in previous studies, there were some differences in the treatment of patients in our center. Some patients who completed their planned chemotherapy were administered oral antivascular targeted drugs. Although we did not find a significant difference between these two groups of patients based on whether to use targeted drugs or not, there was still a tendency of benefit for the group with targeted drugs. Since there were only a small number of patients in this study, the role of oral antivascular drugs on lung cancer patients with SVCS still needs to be explored further. Additionally, the question of whether radiotherapy should be administered after endovascular stenting also needs further investigation.

In conclusion, SVC stenting is now a well‐developed, mature treatment method. When used in conjunction with other modalities as the first‐line treatment for patients with SVCS secondary to lung cancer, it can significantly alleviate symptoms while causing fewer complications. Therefore, SVC stenting plays an important role in the treatment of patients with SVCS secondary to lung cancer.

## Disclosure

The authors declare that they have no competing interests.
